# Neurotoxicity of bupivacaine and liposome bupivacaine after sciatic nerve block in healthy and streptozotocin-induced diabetic mice

**DOI:** 10.1186/s12917-020-02459-4

**Published:** 2020-07-17

**Authors:** Liljana Markova, Nejc Umek, Simon Horvat, Admir Hadžić, Max Kuroda, Tatjana Stopar Pintarič, Vesna Mrak, Erika Cvetko

**Affiliations:** 1 Department of Anaesthesiology and Surgical Intensive Therapy, University Medical Centre Ljubljana, Zaloška cesta 7, 1000 Ljubljana, Slovenia; 2 Institute of Anatomy, Faculty of Medicine, University of Ljubljana, Korytkova ulica 2, 1000 Ljubljana, Slovenia; 3 Department of Animal Science, Biotechnology and Immunology, Biotechnical Faculty, University of Ljubljana, Groblje 3, 1230 Domžale, Slovenia; 4 NYSORA, 2581, Broadway, New York, NY 10025 USA; 5 Ziekenhuis Oost-Limburg, Schiepse Bos 6, Genk, 3600 Belgium

**Keywords:** Bupivacaine hydrochloride, Diabetes, Liposome bupivacaine injectable suspension, Neurotoxicity, Peripheral neuropathy

## Abstract

**Background:**

Long-acting local anaesthetics (e.g. bupivacaine hydrochloride) or sustained-release formulations of bupivacaine (e.g. liposomal bupivacaine) may be neurotoxic when applied in the setting of diabetic neuropathy. The aim of the study was to assess neurotoxicity of bupivacaine and liposome bupivacaine in streptozotocin (STZ) - induced diabetic mice after sciatic nerve block. We used the reduction in fibre density and decreased myelination assessed by G-ratio (defined as axon diameter divided by large fibre diameter) as indicators of local anaesthetic neurotoxicity.

**Results:**

Diabetic mice had higher plasma levels of glucose (*P* < 0.001) and significant differences in the tail flick and plantar test thermal latencies compared to healthy controls (*P* < 0.001). In both diabetic and nondiabetic mice, sciatic nerve block with 0.25% bupivacaine HCl resulted in a significantly greater G-ratio and an axon diameter compared to nerves treated with 1.3% liposome bupivacaine or saline (0.9% sodium chloride) (*P < 0.01*). Moreover, sciatic nerve block with 0.25% bupivacaine HCl resulted in lower fibre density and higher large fibre and axon diameters compared to the control (untreated) sciatic nerves in both STZ-induced diabetic (*P < 0.05*) and nondiabetic mice (*P < 0.01*). No evidence of acute or chronic inflammation was observed in any of the treatment groups.

**Conclusions:**

In our exploratory study the sciatic nerve block with bupivacaine HCl (7 mg/kg), but not liposome bupivacaine (35 mg/kg) or saline, resulted in histomorphometric indices of neurotoxicity. Histologic findings were similar in diabetic and healthy control mice.

## Background

The incidence of diabetes has been steadily increasing, and in the next two decades it is estimated that over 640 million people worldwide will be affected [1]. Although only 10% of patients with diabetes report symptomatic peripheral neuropathy, as many as 50% may already have subclinical neuropathy [2]. Patients with diabetes require surgical procedures more frequently than healthy patients, and due to their comorbidities, peripheral nerve blocks are often recommended as an alternative to general anaesthesia, particularly for lower extremity surgery [3]. In diabetic rats, a prolonged application of high doses of local anaesthetics perineurally has been associated with neurotoxicity [4–6]. In humans, it is not well-established if nerve blocks can exacerbate a pre-existing diabetic neuropathy [7].

Extended-release local anaesthetic formulations have been recently developed to increase the duration of peripheral nerve blocks and to reduce the risk of systemic or local tissue toxicity [8]. Bupivacaine liposome injectable suspension (DepoFoam bupivacaine, EXPAREL®, Pacira Pharmaceuticals, Inc., San Diego, CA, USA) is an extended-release formulation of bupivacaine encapsulated in multivesicular liposomes that has been approved by the U.S. Food and Drug Administration for wound infiltration and interscalene brachial plexus block [9, 10]. Studies to date have found no evidence of neurotoxicity of liposome bupivacaine used for peripheral nerve blocks or epidural applications in animals and humans [11–17]. However, long-acting local anaesthetics (e.g. bupivacaine HCl) or sustained-release formulations of bupivacaine (e.g. liposome bupivacaine) could prove neurotoxic in the presence of a pre-existing neuropathy [5]. The aim of this study was to assess the neurotoxic effects of liposome bupivacaine and bupivacaine hydrochloride (HCl) following perineural injection for sciatic nerve block in streptozotocin (STZ)-induced diabetic mice. We hypothesized that perineural injections of bupivacaine HCl and liposome bupivacaine would result in a reduction of fibre density and decreased myelination in diabetic nerve.

## Results

### Animal characterization

Prior to induction of diabetes, no significant difference in mean body mass was observed between the diabetic [24.6 (1.5) g] and nondiabetic groups [24.7 (2.0) g]. Four weeks after STZ treatment, a lower mean body mass [21.3 (1.9) g] was recorded in diabetic compared to nondiabetic group [27.7 (2.1) g], (*P* < 0.001). At the same time, fasting glucose levels were higher in diabetic [32.4 (2.0) mmol l^− 1^] compared to nondiabetic mice [6.8 (0.9) mmol l^− 1^] (*P* < 0.001).

**Fig. 1:**
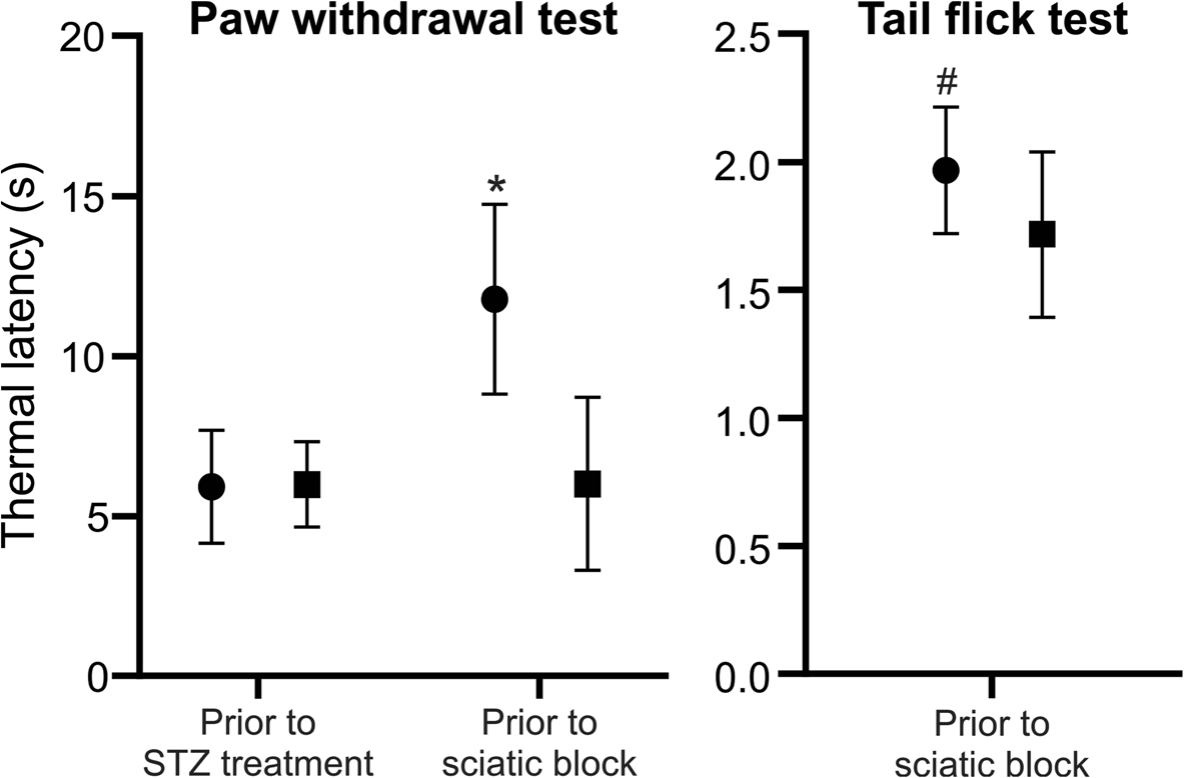
Paw withdrawal test before streptozotocin (STZ) treatment and two days prior to sciatic nerve block and tail flick test two days prior to sciatic nerve block in STZ-induced diabetic (●) (*n* = 18) and nondiabetic (■) (*n* = 18) mice. *P < 0.0001 vs. nondiabetic mice prior to sciatic block and diabetic and nondiabetic mice prior to STZ treatment (one-way ANOVA); #*P* < 0.001 vs. nondiabetic mice (independent *t*-test)

Before application of STZ, no differences were noted in paw withdrawal test thermal latencies between the groups. By contrast, following the STZ treatment and prior to sciatic nerve block, significant differences were observed in tail flick and plantar test thermal latencies between the groups (Fig. 1). The success of sciatic nerve block was confirmed in all animals using a paw withdrawal test.

#### Histopathological evaluations

**Fig. 2:**
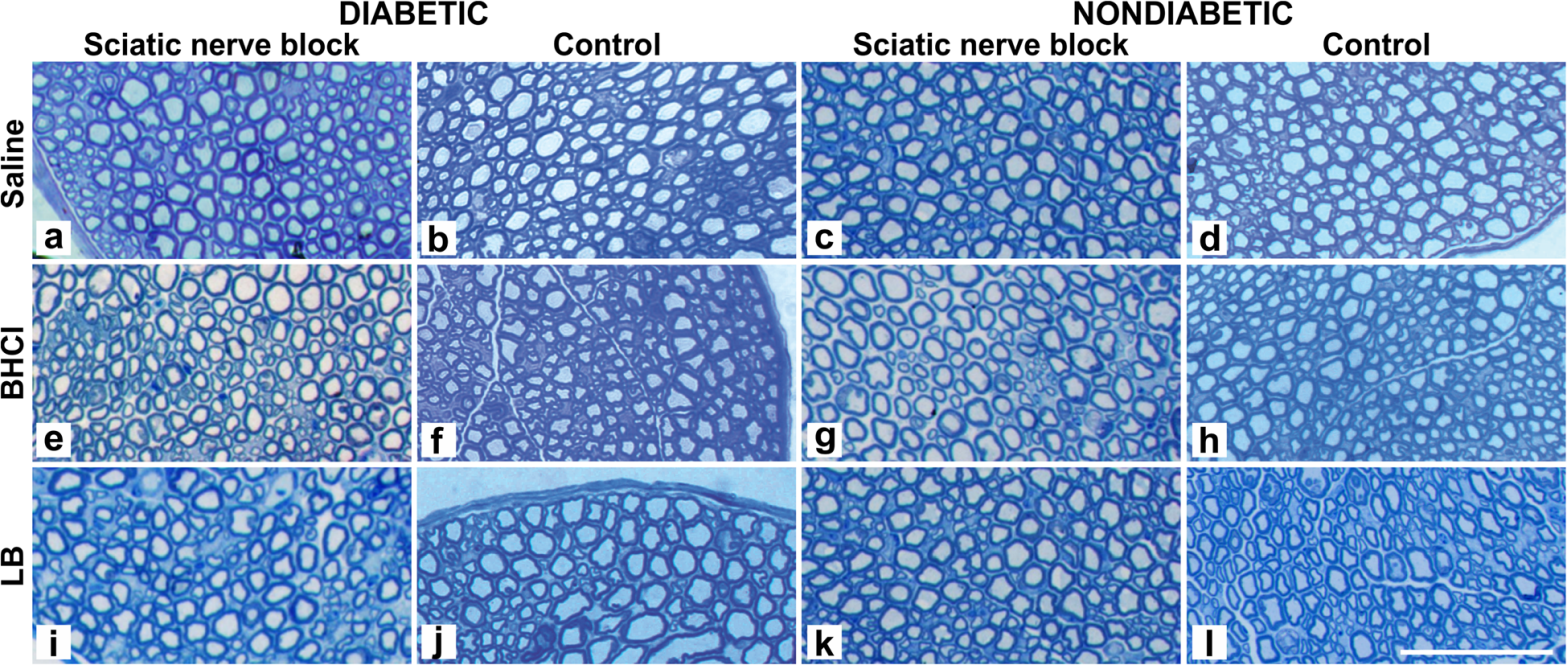
Cross section of the right sciatic nerve seven days after administration of saline, 0.25% bupivacaine hydrochloride (BHCl) and 1.3% liposome bupivacaine (LB) at the sciatic nerve in streptozotocin (STZ)-induced diabetic (**a, e, i**) and nondiabetic mice (**c, g, k**). Untreated nerve from the left leg served as controls (**b, f, j, d, h, l**). Staining with toluidine blue. Bar – 50 µm

**Fig. 3:**
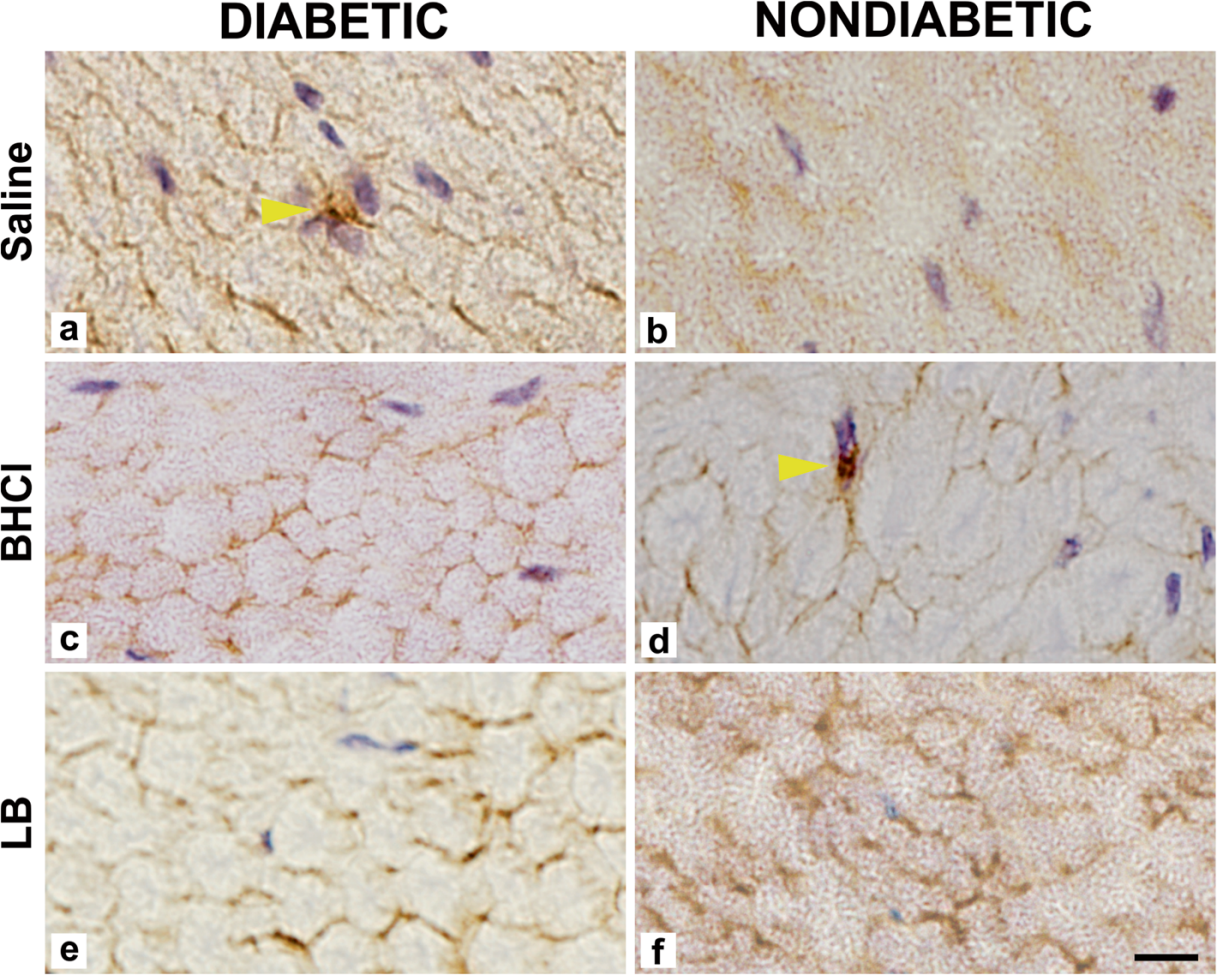
Cross-section of the right sciatic nerve seven days after administration of saline, 0.25% bupivacaine hydrochloride (BHCl) and 1.3% liposome bupivacaine (LB) at the sciatic nerve in STZ-induced diabetic (**a, c, e**) and nondiabetic mice (**b, d, f**) demonstrating rare leucocyte (arrows) infiltration. Immunoreactivity for CD45 is presented. Bar – 50 µm

For each animal, the treated and untreated sciatic nerve tissue specimens were analysed. Data are presented in Table 1. After bupivacaine HCl treatment, the sciatic nerves of diabetic and nondiabetic mice showed a significantly lower fibre density compared to the control (untreated) sciatic nerves, while lower myelin width, and higher axon and large fibre diameters was observed compared to the saline treated nerves. After liposome bupivacaine and saline treatments, by contrast, no differences were observed in morphometric parameters compared to untreated control nerves in both diabetic and nondiabetic mice. Thus, the presence of diabetes did not affect the severity of morphometric changes among the groups (Fig. 2). There was also no evidence of inflammation observed in any specimen; inflammatory cells were scarce, occurring only as discrete leucocytes in a few specimens (Fig. 3).

**Table 1:** Histomorphometric parameters of the sciatic nerve after treatment with bupivacaine hydrochloride (BHCl), liposome bupivacaine (LB) and saline in STZ-induced diabetic mice and nondiabetic control mice

		Treated nerves			Control nerves		
		Saline	BHCl	LB	Saline	BHCl	LB
Fibre density (mm^− 2^)	Diabetic	23724 (6888)	22862 (4349)^†^	30259 (8511)	26232 (4743)	31717 (8258)	23347 (2872)
	Nondiabetic	22492 (4881)	21582 (6144)^††^	21983 (3408)	25969 (2950)	29522 (3558)	25906 (5004)
Large fibre area per total area	Diabetic	65.20 (2.54)	62.63 (7.00)	64.03 (2.50)	67.93 (1.39)	64.58 (3.00)	67.64 (2.00)
	Nondiabetic	66.91 (3.61)	63.95 (6.46)	65.71 (2.53)	65.87 (2.39)	65.19 (2.86)	67.72 (1.90)
Large fibre diameter (µm)	Diabetic	5.58 (0.65)	5.58 (0.54)^†^	4.97 (0.72)	5.37 (0.48)	4.81 (0.68)	5.62 (0.29)
	Nondiabetic	5.82 (0.69)	5.8 (0.75)^††^	5.68 (0.42)	5.27 (0.38)	4.94 (0.21)	5.42 (0.59)
Axon diameter (µm)	Diabetic	3.13 (0.41)	3.53 (0.31)^# ††^	2.79 (0.68)	2.97 (0.55)	2.85 (0.31)	3.36 (0.32)
	Nondiabetic	3.26 (0.49)	3.55 (0.50)^††^	3.25 (0.17)	2.98 (0.09)	2.78 (0.16)	3.04 (0.27)
Myelin width (µm)	Diabetic	1.23 (0.18)	1.01 (0.17)*	1.09 (0.06)	1.14 (0.12)	1.06 (0.19)	1.14 (0.09)
	Nondiabetic	1.28 (0.19)	1.12 (0.29)*	1.21 (0.22)	1.15 (0.20)	1.08 (0.07)	1.19 (0.20)
G-ratio (axon diameter/large fibre diameter)	Diabetic	0.56 (0.03)	0.63 (0.04)** ^##^	0.56 (0.06)	0.55 (0.06)	0.60 (0.09)	0.60 (0.04)
	Nondiabetic	0.56 (0.04)	0.62 (0.06)** ^##^	0.58 (0.05)	0.57 (0.04)	0.56 (0.02)	0.56 (0.03)

Values are means (SD), *n* = 6 for each study group. From two-way ANOVA: *P < 0.05, **P < 0.01 for BHCl- versus saline-treated nerves, and #*P* < 0.05, ##*P* < 0.01 for BHCl- versus LB-treated nerves. From dependent t test: †*P* < 0.05, †† *P* < 0.01 between treated and control nerves

## Discussion

In our study in mice, the sciatic nerve block with bupivacaine HCl, but not liposome bupivacaine, reduced the nerve fibre density and increased the G-ratio, suggestive of demyelinating neuropathy. Under the conditions of our study, the presence of STZ-induced diabetic neuropathy did not appear to affect the severity of pathophysiological changes of nerves treated with bupivacaine HCl or liposome bupivacaine.

Previous studies in healthy rats reported no histologic changes indicative of neurotoxicity after application of bupivacaine HCl and liposome bupivacaine [11, 13, 16]. Both local anaesthetic formulations were also reported to be safe for use in brachial plexus nerve block in rabbits and dogs [12], and in sciatic nerve block in pigs [14]. Similarly, a summary of clinical trials of off-label liposome bupivacaine use for peripheral nerve block in 335 healthy patients without neuropathy concluded that liposome bupivacaine had a similar safety and side effect profile to bupivacaine HCl and saline [15].

However, prolonged exposure of neuropathic nerves to long-acting (bupivacaine) or prolonged-release local anaesthetic formulations (liposome bupivacaine) may result in neurotoxicity. Peak local anaesthetic concentration may also play a role in nerve injury in subjects with diabetic neuropathy [12]. In STZ-induced diabetic rats, application of 2% and 4% but not 1% lidocaine resulted in nerve oedema, degeneration and demyelination of myelinated nerve fibres. [18]. These data led to the teaching that the risk for local anaesthetic-induced nerve injury could be higher in animals and subjects with diabetic neuropathy [4]. Myelin sheet thinning was documented after application of 0.5% ropivacaine, 1% lidocaine with clonidine, and 1% lidocaine with epinephrine in STZ-induced diabetic rats [5]. Moreover, application of 2% lidocaine may also be a cause of neurotoxicity in obese diabetic rats even with subclinical diabetic neuropathy [19]. The duration of the local anaesthetics exposure could also contribute to neurotoxicity [5].

The available reports on the neurotoxic effects of local anaesthetic [20–22] suggest that neurotoxicity is not consistent and may depend on the model, mode of application, type of local anaesthetic and other factors. With liposome bupivacaine, however, the delayed release of free bupivacaine from the selected dose of liposome bupivacaine in our study may not have reached the local tissue concentration level capable of causing neurotoxicity [23]. The pharmacokinetic studies indicate that the release of free bupivacaine from liposome bupivacaine is not linear; the bupivacaine releases only after 12 h. However, since the free bupivacaine release occurs over 72 h, its local tissue concentration may be too small to cause neurotoxicity. Indeed, the pharmacokinetic data from clinical studies indicate that the release of the local anaesthetic from the formulation is low, resulting in light sensory, and no motor block [24].

Diabetic neuropathic nerves exhibit complex functional changes [7]. In our study, diabetic mice showed early functional sensory impairment without morphological correlates, consistent with previous findings in STZ-induced diabetic rats [5, 25, 26]. In contrast, in 6-week-old male STZ-induced diabetic mice of the same strain used in another study, thin, disorganized and demyelinated sciatic nerve fibres were observed [27]. Given that axon and myelin sheet growth are not yet completed in the 6 week old mice [28], the difference in animal age at the time of STZ application (6 weeks in Pan et al. [27] and 8 weeks in our study), may be responsible for the differential effects of STZ and hyperglycaemia on the sciatic nerves.

In our study, the number of small sciatic nerve fibres was lower following bupivacaine HCl compared to saline application in nondiabetic mice. However, this effect was not replicated in other morphometric studies after perineural bupivacaine HCl application in rat, rabbit or dog model [11–13]. Given that the small nerve fibres are usually first affected by diabetes [29], bupivacaine HCl may cause small nerve fibre degeneration after nerve blockade in the setting of diabetic neuropathy.

We did not find any signs of inflammation in diabetic and nondiabetic nerves, which is consistent with the observations reported by McAlvin et al. [13]. In contrast, using an open approach for sciatic nerve block in nondiabetic rats, infiltrations with macrophages, lymphocytes and fibroblasts have been observed after both liposome bupivacaine and bupivacaine HCl injections [11].

Our results should not be directly extrapolated to the clinical practice of perineural application of bupivacaine and liposome bupivacaine, due to a number of limitations. First, being the first study on nerve blocks in diabetic mice with bupivacaine and liposome bupivacaine, we used a single, exploratory dose and concentration. In addition, the observed changes in our study may be specific to our animal model and nerve block technique. We used a percutaneous block technique to minimize nerve inflammation due to the procedure [5]. Furthermore, inflammatory changes in a STZ-induced diabetic model may be diminished, because STZ depletes immune cells in the peripheral nervous system up to 3 weeks after treatment [30]. A longer-term longitudinal study in a high-fat-diet-induced diabetes type-2 model could be more informative in assessing possible inflammatory effects.

The unifascicular sciatic nerve in mice may be more sensitive to neurotoxic effects compared to multifascicular nerves with abundant connective tissue within epineurium in humans. Although large animal models may better resemble the multifascicular sciatic nerve seen in humans, there are difficulties in establishing diabetes and diabetic neuropathy in larger animals [31]. Further, the STZ-induced diabetic mouse model does not correlate well with all aspects of type-1 or type-2 diabetes in humans [30]. High-fat-diet-induced diabetes is thought to better represent the more prevalent type-2 diabetes in humans; however, there is no diabetic mouse model that intimately mirrors the human pathophysiology of diabetes [32]. And finally, given that only females were used in our study, another study in males is warranted as sex differences in STZ sensitivity have been noted in rodent models [33]. Our study should be viewed as an exploratory or a pilot experiment. However, we believe that our data could be useful to inform future studies and investigators in structuring more robust, dose-ranging studies on neurotoxicity in diabetic mice.

## Conclusions

Under the conditions of our study, the preliminary data suggest that application of bupivacaine HCl, but not liposome bupivacaine, resulted in histological evidence of neurotoxicity in both STZ-induced diabetic and nondiabetic mice.

## Methods

The study was carried out in accordance with the recommendations of the Guide for the Care and the Use of Laboratory Animals of the National Institutes of Health (National Research Council (U.S.) [34], the Committee for the Update of the Guide for the Care and Use of Laboratory Animals, and the Institute for Laboratory Animal Research (U.S.), 2011). The study was approved by the Ethical Committee for laboratory animals of the Republic of Slovenia (Permit Number: U34401-21/2013/6) following European directives on the use of laboratory animals in research and the ARRIVE guidelines.

### Animal housing and induction/confirmation of diabetes

Six weeks old C57BL/6J-OlaHsd female mice (*n* = 36, weight 25–30 g) were obtained from Harlan Laboratories – Envigo (Italy) and reared at the Centre for Laboratory Animals of the Biotechnical Faculty of the University of Ljubljana. All mice were housed individually in ventilated cages (IVC system) with temperature maintained at 23 ± 1° C, humidity maintained at 40–60%, and a 12-hour light/12-hour dark cycle.

At the age of 8 weeks, after 2 weeks of quarantine and acclimatization period with free access to clean water and standardized diet (Mucedola, Milan, Italy), diabetes type 1 was induced in mice by intraperitoneal injection of 200 mg kg^− 1^ STZ in accordance with the protocols for achieving STZ-induced diabetes in mice [27, 35]. STZ is an alkylating agent that induces degeneration in pancreatic β islets [18, 36]. Diabetes was confirmed by measuring a fasting glucose level using Bayer Contour glucose meter (Ascensia Diabetes Care Holdings AG, Switzerland) three weeks after STZ injection. Animals with a fasting glucose level of more than 25 mmol l^− 1^ were considered diabetic, while those with less than 8 mmol l^− 1^ were considered nondiabetic [27]. All STZ induced diabetic mice included in the study met the criteria for diabetes.

#### Verification of diabetic neuropathy

To confirm the presence of peripheral sensory neuropathy, tail flick and paw withdrawal tests were performed using Combination Plantar/Tail Flick Analgesia Meter (IITC Life Science, California, USA) with infrared intensity set at 40% and 50%, and cut-off times of 4.00 and 15.00 s, respectively [37]. The paw withdrawal test was performed two days before the STZ application and two days prior to the sciatic nerve block, whereas the tail flick test was performed two days prior to the sciatic nerve block. Heat stimulation was repeated 3 times at 5 min-intervals; the mean value of the two measurements was used as the baseline [38]. The plantar method is based on Hargreaves method of quantifying the heat thresholds in the hind paws of rodents upon application of radiant or infrared heat stimulus [39]. The tail flick test involved the application of a heat stimulus to the tail after which the time for the tail to ‘‘flick’’ or twitch was recorded. We used a tail temperature option with an automatic temperature trigger at the start of the tests. Once the pre-set temperature was reached, the timer was automatically triggered and stopped after the tail flicks and the light had stopped. The automatic readouts of the start and end temperatures, and the test time improved a repeatability of the measurements. This option has solved the problem associated with “tail temperature prior to and at the end of testing” [40]. While the recent reviews discussed advantages as well as disadvantages of both tests, the two methods are still considered as relevant stimulus-evoked nociception tests [41].

### Study groups

Eighteen STZ-induced diabetic and eighteen nondiabetic mice were randomized into the three treatment groups. According to the group assignment, both diabetic and nondiabetic groups included 6 mice treated with 35 mg kg^− 1^ 1.3% liposome bupivacaine (EXPAREL), 6 mice treated with 7 mg kg^− 1^ 0.25% bupivacaine HCl (AstraZeneca UK Ltd, UK), and 6 mice treated with saline (NaCl Braun, 9 mg ml^− 1^ injection solution, B Braun Melsungen AG, Germany).

### Sciatic nerve block

The mice were anaesthetized with isoflurane up to 4% in a nitrous oxide/oxygen mixture (N2O/O2) via a face-mask. Sciatic nerve blocks were performed by injecting local anaesthetics or saline perineurally using a 29-gauge needle (Omnican®A, B. Braun Melsungen AG, Germany) while held in a lateral recumbent position with paws in a right angle with the trunk. The needle was introduced posteromedially towards the greater trochanter in an anteromedial direction. After encountering the ischial tuberosity, 85 µl of testing solution was injected by a single trained research staff member, blinded to the study group assignment [13, 42, 43]. The success of the sciatic nerve block was evaluated 20 min after using the paw withdrawal test.

### Histopathological evaluation of the sciatic nerve

The animals were sacrificed by cervical dislocation one week after the nerve block in order to allow enough time for nerve pathohistological changes to manifest [44]. At the site of local anaesthetic injection and contralaterally, five mm-long sections of the sciatic nerve were harvested and processed for Epon-embedding for histomorphometric evaluations. After initial fixation in Karnovsky’s KII Solution (2.5% glutaraldehyde, 4.0% paraformaldehyde in 0.1 M sodium cacodylate buffer, pH 7.4), the nerve sections were post-fixed in an 1:1 solution of 2% aqueous osmium tetroxide and 3% potassium ferrocyanide. Dehydration was accomplished with graded ethanol solutions and propylene oxide following Epon embedding. A high-resolution light microscope (Eclipse E800; Nikon, Tokyo, Japan) was used to study the prepared 0.5 µm toluidine blue stained cross-sections with images captured by a digital camera (DXM1200F™, Nikon, Tokyo, Japan) connected to the microscope. Images were analysed by a single operator blinded to group assignment.

Morphometric analysis was performed using the Ellipse program (ViDiTo, version 2.0.7.1, 2004, Košice, Slovakia) [14]. Randomly selected areas of the nerve were analysed. The outer border of the nerve fibres and the inner border of the myelin sheaths were assessed at high magnification followed by measurement of the nerve fibre density, proportion of large fibres (percent of fibres where the myelin sheet is visible and can be circumscribed), large fibre diameter, axon diameter and myelin width. Furthermore, G-ratio defined as axon diameter divided by large fibre diameter of the myelin sheath was also calculated [45]. The images were analysed by a trained evaluator blinded to group assignment.

Histopathological evaluation was also employed to assess inflammatory cell infiltration in the histological specimens. Frozen samples of the sciatic nerve were sliced into 10 µm transverse sections processed for immunohistochemistry for leucocyte receptor-type tyrosine-protein phosphatase C (CD45) labelling with anti-CD45 antibody (MCA1388, Bio-Rad Laboratories Inc., San Francisco, CA, USA) and revealed by a secondary antibody P0260 (Dako, Glostrup, Denmark). Positive and negative tissue controls were included with each batch of slides as a check on correct tissue preparation and staining techniques. Sections of mouse thymus served as positive control for the presence of leukocytes. For negative controls, the sections in which the primary antibody was replaced with phosphate-buffered saline were used. The images were analysed by a trained evaluator blinded to group assignment.

### Statistical analysis

The Shapiro-Wilk test was used to evaluate the groups for normality. If normality and equal variance assumptions were met, differences in histomorphometric parameters among treatment groups were tested by two-way analysis of variance (ANOVA) followed by Bonferoni *post-hoc* tests that corrected the *p*-values for the subgroup analyses. The dependent *t*-test for paired samples was used to test differences in histomorphometric parameters between treated and untreated sciatic nerves in the same animal. One-way ANOVA, followed by Tukey post-hoc tests was used for paw withdrawal test. Independent *t*-test was used to compare tail flick test results, body mass and fasting glucose. Statistical analysis was performed with the IBM SPSS Statistics for Windows, version 25 (IBM Corp, NY, USA). Differences were deemed statistically significant at *P* < 0.05. Data are presented as means (standard deviation).

The sample size calculation was based on the primary research hypothesis that the STZ-induced diabetic and nondiabetic nerves would differ in their fibre density as an indicator of local anaesthetic neurotoxicity [46]. Using the difference in mean fibre density (14,000 fibres per mm^2^), pooled standard deviation (1600 fibres per mm^2^), Type I alpha (0.01), and a desired power (0.90), the sample size was estimated at 6 animals in each treatment group for this two-sided test of a completely crossed 2 × 3 ANOVA (diabetic/nondiabetic by liposome bupivacaine/bupivacaine HCl/saline).

## Data Availability

The datasets used and/or analysed during the current study are available from the corresponding author on reasonable request.

## Abbreviations

ANOVA: Analysis of variance
BHCL: Bupivacaine hydrochloride
HCl: Hydrochloride
IVC: Individually ventilated cages
LB: Liposome bupivacaine
STZ: Streptozotocin
